# The development of a 16S rRNA gene based PCR for the identification of *Streptococcus pneumoniae *and comparison with four other species specific PCR assays

**DOI:** 10.1186/1471-2334-10-104

**Published:** 2010-04-29

**Authors:** Nabil Abdullah El Aila, Stefan Emler, Tarja Kaijalainen, Thierry De Baere, Bart Saerens, Elife Alkan, Pieter Deschaght, Rita Verhelst, Mario Vaneechoutte

**Affiliations:** 1 Laboratory Bacteriology Research, Department of Chemistry, Microbiology and Immunology, University of Ghent, Ghent, Belgium; 2 SmartGene, Zug, Switzerland; 3 National Reference Laboratory for Pneumococcus, National Institute for Health and Welfare (THL), Oulu, Finland; 4 Scientific Institute of Public Health, Brussels, Belgium

## Abstract

**Background:**

*Streptococcus pneumoniae *is one of the most frequently encountered pathogens in humans but its differentiation from closely related but less pathogenic streptococci remains a challenge.

**Methods:**

This report describes a newly-developed PCR assay (Spne-PCR), amplifying a 217 bp product of the 16S rRNA gene of *S. pneumoniae*, and its performance compared to other genotypic and phenotypic tests.

**Results:**

The new PCR assay designed in this study, proved to be specific at 57°C for *S. pneumoniae*, not amplifying *S. pseudopneumoniae *or any other streptococcal strain or any strains from other upper airway pathogenic species. PCR assays (psaA, LytA, ply, spn9802-PCR) were previously described for the specific amplification of *S. pneumoniae*, but *psaA*-PCR was the only one found not to cross-react with *S. pseudopneumoniae*.

**Conclusion:**

Spne-PCR, developed for this study, and psaA-PCR were the only two assays which did not mis-identify *S. pseudopneumoniae *as *S. pneumoniae*. Four other PCR assays and the AccuProbe assay were unable to distinguish between these species.

## Background

*Streptococcus pneumoniae *is one of the most pathogenic bacteria involved in human disease [1], causing bronchitis, pneumonia, as well as life-threatening meningitis and bloodstream infections [2]. Culture-based methods are usually applied to detect *S. pneumoniae *from patient samples and to differentiate it from other less pathogenic viridans streptococci, frequently encountered in respiratory samples. Differentiation is also important with regard to resistance testing, since different antibiotic susceptibility breakpoints are applied for *S. pneumoniae *with regard to other viridans species [3]. Culture-based identification methods usually rely on optochin susceptibility, agglutination and bile-solubility, sometimes confirmed by specific probes (Accuprobe™, Genprobe) [4].

However, straightforward phenotypic identification of pneumococci is hampered by the occurrence of optochin resistant *S. pneumoniae *variants [5-8] and. In addition, closely related *S. pseudopneumoniae *is difficult to distinguish from *S. pneumoniae *and is e.g. positive with AccuProbe as well [4].

It is of clinical relevance to rapidly and specifically detect *S. pneumoniae*. Therefore several PCR assays have been developed over the past decades [9-14].

This study compared the specificity of four published *S. pneumoniae *PCR assays to that of a new approach, based on the 16S rRNA gene. One novelty of this approach regards the in-silico design of specific primers using published *Streptococcus *sequences, filtered for quality and annotation-reliability by profile-based methods (SmartGene, Zug, Switzerland). The approach used by this program relies on a systematic analysis of all published 16S rRNA gene sequences for all streptococci (and closely related organisms), using sequence profiles to eliminate obviously incomplete or erroneous submissions, which could induce wrong alignments.

Sequences with a likely incorrect annotation (shown by low match scores to other sequences of the same species), with unexpected deletions/insertions, or with non useful annotations (e.g. "uncultured") were excluded, since they could induce misleading alignments. Thus, the most representative 16S rRNA sequences were determined for each species. Such a database of representative sequences was used to align closely related streptococcal species to identify specific positions in the 16S rRNA gene for the purpose of species-specific identification. Once these positions were identified, general searches on relevant published sequences of *S. pneumoniae *and closely related relatives confirmed the consistency of the sequence pattern found. This method helped to detect discriminative species-specific sequence patterns for *S. pneumoniae *and *S. pseudopneumoniae*, thus saving time and effort through reduction of non-specific results in wet-lab testing.

## Methods

### Bacterial strains

A total of 73 streptococcal strains were analyzed in this study, as listed in Table 1, i.e. 8 reference strains of *Streptococcus mitis *[11] and seven reference strains of *S. oralis *[11], including three reference strains and the type strain; 19 strains of *S. pneumoniae*, including two reference strains and the type strain and including 10 optochin resistant strains, for which it was concluded in a previous study [11] that these were genuine *S. pneumoniae*. In addition, a total of 30 optochin resistant pneumococcus-like streptococci already well-characterized in an earlier study [11], were also included. Finally, the type strain, two reference strains and one clinical strain of *S. pseudopneumoniae *[4] were included. A total of 12 isolates belonging to the species *Haemophilus influenzae *(NCTC 8143^T^), *Moraxella catarrhalis *(ATCC 25238^T ^and clinical isolate VG S86 0025), *Staphylococcus aureus *(ATCC 29213 and NCTC 08530), *S. epidermidis *(CCM2 124^T ^and CNRS N860069), *Streptococcus agalactiae *(LMG 14694^T^), *S. anginosus *(LMG 14502^T^), *S. gallolyticus *(LMG 16802^T^), *S. mutans *(LMG 14558^T^) and *S. pyogenes *(LMG 14237), i.e. species also present in the upper airway tract and/or other *Streptococcus *species, were used to test the specificity of primer set Spne1-Spne2Rb. In addition, *S. parasanguinis *(LMG 14537^T ^and LMG 14538) and *S. sanguinis *(LMG 14656, LMG 14657 and LMG 14702^T^) isolates were used to test the specificity of primer set Spne1-Spne2Rb.

**Table 1:** Species and strains studied, phenotypic characteristics, and results for PCR assays

Species^a^	Strain Number	Original Number^b^	Spne-PCR	*psaA*-PCR	*lytA*-PCR	*ply*-PCR	*spn9802*-PCR
*Streptococcus mitis*	STR025	LMG 14557^T^	-	-	-	-	-
*Streptococcus mitis*	STR056	LMG 14552	-	-	-	-	-
*Streptococcus mitis*	STR226	94 03 0728	-	-	-	-	-
*Streptococcus mitis*	STR227	94 04 0401	-	-	-	-	-
*Streptococcus mitis*	STR228	97 03 2943	-	-	-	-	-
*Streptococcus mitis*	STR229	98 05 5898	-	-	-	-	-
*Streptococcus mitis*	STR230	98 07 1207	-	-	-	-	-
*Streptococcus mitis*	STR231	98 09 0066	-	-	-	-	-
*Streptococcus oralis*	STR024	LMG 14553	-	-	-	-	-
*Streptococcus oralis*	STR028	LMG 14532^T^	-	-	-	-	-
*Streptococcus oralis*	STR029	LMG 14533	-	-	-	-	-
*Streptococcus oralis*	STR030	LMG 14534	-	-	-	-	-
*Streptococcus oralis*	STR232	94 08 5574	-	-	-	-	-
*Streptococcus oralis*	STR233	98 05 5050	-	-	-	-	-
*Streptococcus oralis*	STR234	98 10 1512	-	-	-	-	
*Streptococcus parasanguinis*	STR031	LMG 14537^T^	-	NT	NT	NT	NT
*Streptococcus parasanguinis*	STR032	LMG 14538	-	NT	NT	NT	NT
*Streptococcus sanguinis*	STR038	LMG 14656	-	NT	NT	NT	-
*Streptococcus sanguinis*	STR039	LMG 14657	-	NT	NT	NT	-
*Streptococcus sanguinis*	STR059	LMG 14702^T^	-	NT	NT	NT	NT
*Streptococcus pneumoniae*	STR061	LMG 14545^Tit>^	+	+	+	+	+
*Streptococcus pneumoniae*	STR062	LMG 15155	+	+	+	+	+
*Streptococcus pneumoniae*	STR063	LMG 16738	+	+	+	+	+
*Streptococcus pneumoniae*	STR235	93 08 1310	+	+	+	+	+
*Streptococcus pneumoniae*	STR236	93 09 03230	+	+	+	+	+
*Streptococcus pneumoniae*	STR237	93 09 1111	+	+	+	+	+
*Streptococcus pneumoniae*	STR238	98 10 1630	+	+	+	+	+
*Streptococcus pneumoniae*	STR239	98 10 3326	+	+	+	+	+
*Streptococcus pneumoniae*	STR240	98 10 3367	+	+	+	+	+
*Streptococcus pneumoniae*. Group I: oR C+ A+	STR119	KTL004	+	+	+	+	+
*Streptococcus pneumoniae*. Group I: oR C+ A+	STR120	KTL005	+	+	+	+	+
*Streptococcus pneumoniae*. Group I: oR C+ A+	STR125	KTL013	+	+	+	+	+
*Streptococcus pneumoniae*. Group I: oR C+ A+	STR127	KTL017	+	+	+	+	+
*Streptococcus pneumoniae*. Group I: oR C+ A+	STR141	KTL043	+	+	+	+	+
*Streptococcus pneumoniae*. Group I: oR C+ A+	STR144	KTL051	+	+	+	+	+
*Streptococcus pneumoniae*. Group I: oR C+ A+	STR147	KTL056	+	+	+	+	+
*Streptococcus pneumoniae*. Group I: oR C+ A+	STR149	KTL063	+	+	+	+	+
*Streptococcus pneumoniae*. Group I: oR C+ A+	STR155a	KTL076	+	+	+	+	+
*Streptococcus pneumoniae*. Group I: oR C+ A+	STR164	KTL093	+	+	+	+	+
*Streptococcus pseudopneumoniae*. (Group I: oR C+ A+)	STR157	KTL079	-	-	+	+	+
*Streptococcus pseudopneumoniae*	STR269	CCUG 48465	-	-	+	+	+
*Streptococcus pseudopneumoniae*	STR270	CCUG 49455^T^	-	-	+	+	+
*Streptococcus pseudopneumoniae*	STR271	CCUG 50866	-	-	+	+	+
*Streptococcus *sp. Group IIa: oR C- A+	STR150	KTL065	-	-	+	+	+
*Streptococcus *sp. Group IIa: oR C- A+	STR162	KTL089	-	-	-	+	-
*Streptococcus *sp. Group IIa: oR C- A+	STR165	KTL096	-	-	+	+	-
*Streptococcus *sp. Group IIb: oR C- A-	STR122	KTL007	-	-	-	+	-
*Streptococcus *sp. Group IIb: oR C- A-	STR133	KTL028	-	-	-	+	-
*Streptococcus *sp. Group IIb: oR C- A-	STR130	KTL021	-	-	-	+	-
*Streptococcus *sp. Group IIb: oR C- A-	STR137	KTL035	-	-	-	+	-
*Streptococcus *sp. Group IIb: oR C- A-	STR152	KTL069	-	-	+	+	-
*Streptococcus *sp. Group IIb: oR C- A-	STR153	KTL072	-	-	-	+	-
*Streptococcus *sp. Group IIb: oR C- A-	STR131	KTL022	-	-	+	-	-
*Streptococcus *sp. Group IIb: oR C- A-	STR151 A	KTL068	-	-	-	-	-
*Streptococcus *sp. Group IIc: oR C- A-	STR118	KTL003	-	-	-	-	-
*Streptococcus *sp. Group IIc: oR C- A-	STR121	KTL006	-	-	-	-	-
*Streptococcus *sp. Group IIc: oR C- A-	STR136	KTL034	-	-	-	-	-
*Streptococcus *sp. Group IIc: oR C- A-	STR138	KTL038	-	-	-	-	-
*Streptococcus *sp. Group IIc: oR C- A-	STR145	KTL054	-	-	-	-	-
*Streptococcus *sp. Group IIc: oR C- A-	STR154	KTL073	-	-	-	-	-
*Streptococcus *sp. Group IIc: oR C- A-	STR123	KTL008	-	-	-	-	-
*Streptococcus *sp. Group IIc: oR C- A-	STR128	KTL019	-	-	-	-	-
*Streptococcus *sp. Group IIc: oR C- A-	STR132	KTL023	-	-	-	-	-
*Streptococcus *sp. Group IIc: oR C- A-	STR134	KTL029	-	-	-	-	-
*Streptococcus *sp. Group IIc: oR C- A-	STR139	KTL039	-	-	-	-	-
*Streptococcus *sp. Group IIc: oR C- A-	STR158	KTL081	-	-	-	-	-
*Streptococcus *sp. Group IIc: oR C- A-	STR159	KTL083	-	-	-	-	-
*Streptococcus *sp. Group IIc: oR C- A-	STR156	KTL077	-	-	-	-	-
*Streptococcus *sp. Group IIc: oR C- A-	STR140	KTL041	-	-	-	-	-
*Streptococcus *sp. Group IIc: oR C- A-	STR160	KTL085	-	-	-	-	-
*Streptococcus *sp. Group IIc: oR C- A-	STR161	KTL087	-	-	-	-	-
*Streptococcus *sp. Group IIc: oR C- A-	STR135	KTL030	-	-	-	-	-
*Streptococcus *sp. Group IIc: oR C- A-	STR143	KTL050	-	-	-	-	-

a: oR: optochin resistant, C: capsule, A: AccuProbe.

b: CCUG: Culture Collection of the University of Göteborg, Sweden; KTL: National Public Health Institute, Helsinki, Finland; LMG: LMG, Laboratorium voor Microbiologie, Gent Culture Collection.

NT: not tested

**DNA-extraction **was carried out by alkaline lysis as described previously [15]. always starting from one colony.

### *spn9802*-PCR assay

The amplification reactions were performed as described previously [9], with minor modifications. Briefly, amplification was performed in a reaction mixture of 10 μl, containing 5 μl PCR GoTaqGreen Mix (Promega Benelux, Leiden, the Netherlands), 2 μM of each of the forward primer spn9802-143F and the reverse primer spn9802-304R and 1 μl of the DNA extract. The following thermal cycling profile was applied, using a Veriti™ Thermal Cycler (Applied Biosystems, Foster City, Ca.): initial denaturation at 94°C for 2 min, then 25 cycles consisting of 94°C for 10 sec, 58°C for 15 sec and 72°C for 1 min, followed by a final extension step at 72°C for 5 min. All PCR products were electrophoresed in 2% agarose gels and stained with ethidium bromide.

### Newly developed PCR assay: 16S rRNA gene based Spne-PCR assay

**Figure 1:**
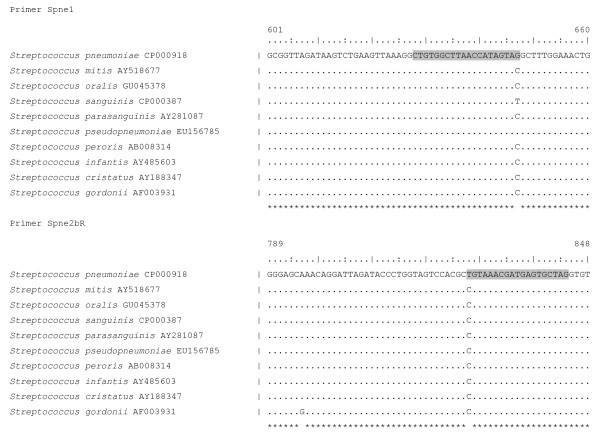
**Sequence and position of the primers, newly developed in this study, specific for amplification of *S. pneumoniae*, and the homologous sequences for the *S. mitis *group species**.

Extensive data-mining using commercial software (Integrated Database Network System IDNS™, SmartGene, Zug, Switzerland), which allows rapid screening of validated published sequences of species of interest against other closely related species was used to design primers for the specific amplification of *S. pneumoniae*. Primers were designed to match exactly 2 positions within the 16S rRNA gene, which allow to distinguish *S. pneumoniae *from *S. pseudopneumoniae *(Figure 1).

**Figure 2:**
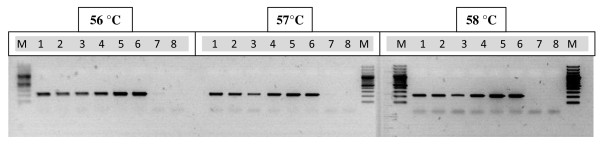
**Gradient Spne-PCR results for six *S. pneumoniae *and two *S. pseudopneumoniae *isolates**. M: marker (100 basepair ladder); lanes 1-6: *S. pneumoniae *isolates STR235 (lane 1), STR236 (lane 2), STR237 (lane 3), STR239 (lane 4), STR144 (lane 5), STR147 (lane 6); lanes 7-8:*S. pseudopneumoniae *isolates STR269 (lane 7) and STR157 (lane 8).

Amplifications were performed on an Applied Biosystems Veriti thermal cycler in reaction mixtures of 10 μl containing 5 μl PCR GoTaqGreen Mix (Promega Benelux, Leiden, the Netherlands), 0.5 μM of each of the two primers and 1 μl of the DNA extract. The 16S rRNA gene primers Spne1-Spne2Rb was designed to amplify *S. pneumoniae*, and the stringent annealing temperature was determined by gradient PCR as 57°C (Figure 2)

The cycling parameters were 94°C for 2 min, followed by 30 cycles of 10 sec at 94°C, 15 sec at 57°C, 1 min at 72°C, and final extension at 72°C for 5 min. The indicated primers are amplifying a 217 bp product of the 16S rRNA gene of *S. pneumoniae*.

**Other PCR assays**, i.e. *ply*- [16], *psaA*- [17] and *lytA*-PCR [18] were described previously [11].

### Sequence analysis

Published streptococcal and other 16S rRNA sequences were extracted from EMBL using proprietary extraction methods based on sequence profiles and annotation searches; representative sequences for each species were determined using a proprietary algorithm developed by SmartGene for bacterial 16S rRNA sequences (SmartGene IDNS™ Bacteria Module). Sequences were analyzed and compared using search and alignment functions of the IDNS™ Bacteria Module of SmartGene. Results were exported as CLUSTAL-A or FASTA files for further analysis.

## Results & Discussion

Previously we reported on encapsulation, AccuProbe hybridization and *psaA*, *lytA *and *ply*-PCR results for a collection of 49 optochin resistant alpha-hemolytic streptococcal isolates, suspected of being atypical pneumococci [11]. We concluded that for some strains identification problems continue to exist, despite the application of combined genotypic and phenotypic tests and we found *psa*A-PCR to be the most specific genotypic technique for the identification of genuine pneumococci and optochin resistant pneumococci. In addition, in this study, 16S rRNA gene based primers Spne1 and Spne2Rb (Spne-PCR) were designed to amplify *S. pneumoniae *isolates and we tested these primers for specific amplification of *S. pneumoniae *using the same, previously well-studied selection of isolates [11], to which three *S. pseudopneumoniae *and two *S. parasanguinis *isolates were added. Also, we tested the specificity of a PCR assay, i.e. *spn9802*-PCR [9], that was described in the meantime for amplification of *S. pneumoniae*.

All five PCR assays were negative for seven commonly found respiratory tract species, for 8 *S. mitis *group isolates, for 7 *S. oralis *isolates and for the 19 optochin R streptococcal isolates for which we had already concluded in the previous study [11] that they were non *S. pneumoniae*. In addition, *Spne*-PCR was negative for the three *S. sanguinis *and the two *S. parasanguinis *isolates.

All five PCR assays were positive for the nine optochin susceptible *S. pneumoniae *isolates included and for the ten optochin resistant streptococci, which had been considered as *S. pneumoniae *already, based on the PCR results from our previous study [11](Table 1).

Thus far, all five PCR assays were found to be equally specific. However, for a total of 11 optochin R streptococcal isolates, designated during the previous study as group IIa and group IIb, four were positive with *lytA*-PCR, nine with *ply*-PCR and Spn9802-PCR (9), whereas none of these isolates yielded a positive result when tested with *psaA*-PCR and *Spne*-PCR.

The *S. pseudopneumoniae *type strain CCUG 49455^T^, the two *S. pseudopneumoniae *reference isolates CCUG 48465 and CCUG 50866, and one optochin-resistant pneumococcus-like isolate (KTL079) were positive with *lytA*-PCR, *ply*-PCR and *spn9802*-PCR, but negative with *Spne*-PCR and *psaA*-PCR. Sequence determination of the 16S rRNA gene (accession number: FJ827123) identified this clinical isolate unambiguously as *S. pseudopneumoniae*. This isolate was also positive with AccuProbe (Table 1).

Several primer sets have been described for the species specific amplification of *S. pneumoniae*. However, in our hands, the primer sets lytA [18,19], ply [16] and spn9208 [20], were found to amplify strains of *S. pseudopneumoniae *as well. Also the commercial AccuProbe hybridization assay yielded a false positive result for the single *S. pseudopneumoniae *isolate that was tested by others [4] and for one strain (KTL079) that was tested by us in our previous study [11].

The PCR-assay *Spne*-PCR, described here, is specific for *S. pneumoniae*, without cross-reactivity to the four *S. pseudopneumoniae *strains tested. The differentiation of *S. pneumoniae *from *S. pseudopneumoniae *is important since the pathogenic potential of *S. pneumoniae *is far higher than that of *S. pseudopneumoniae*. The clinical relevance of *S*. *pseudopneumoniae *has not yet been established, although it may be associated with chronic obstructive pulmonary disease [21].

In addition, the advantage of a specific PCR test on the basis of the 16S rRNA gene is that there are several copies of this gene, i.e. 5 to 6 in other *Streptococcus *species [22] and 4 copies in the fully sequenced genome of *S. pneumoniae *R6 (AE007317), thus possibly enhancing sensitivity when this PCR is applied for the direct detection and identification of *S. pneumoniae *in clinical samples.

In addition, 16S rRNA gene sequencing is a standard method in microbial taxonomy and can be applied directly on the amplified products of this PCR assay to to help resolve potentially ambiguous results.

## Conclusions

*Spne*-PCR, described here, and *psaA*-PCR [17] were the only two out of 5 PCR assays tested, which did not misidentify *S. pseudopneumoniae *as *S. pneumoniae*. The approach using representative sequences rather than unfiltered data from Genbank enabled us to select the correct sites for reliable species differentiation out of less relevant and consistent variations and allowed us to design highly specific primers. Future studies should enable us to develop an assay specifically for *S. pseudopneumoniae *and a real-time, multiplex assay for rapid discrimination of the most important viridans streptococci in bacterial cultures or patient samples.

## Competing interests

The authors declare that they have no competing interests.

## Authors' contributions

NAE, SE, RV and MV participated in the development of the study design, the analysis of the study samples, the collection, analysis and interpretation of the data, and in the writing of the report. TK, TDB, BS, EA and PD participated in the analysis of the study samples and interpretation of the data. All authors read and approved the final manuscript.

## Pre-publication history

The pre-publication history for this paper can be accessed here:

http://www.biomedcentral.com/1471-2334/10/104/prepub
